# Draft genome sequence of *Agathobacter rectalis* H10.1 isolated from the feces of a child with ulcerative colitis in remission

**DOI:** 10.1128/mra.00920-25

**Published:** 2025-11-24

**Authors:** Sasanka Weerasingha, David R. Mack, Alain Stintzi

**Affiliations:** 1 School of Pharmaceutical Sciences, Faculty of Medicine, University of Ottawa 6363, Ottawa, Ontario, Canada; 2 Department of Biochemistry, Microbiology and Immunology, Faculty of Medicine, University of Ottawa 6363, Ottawa, Ontario, Canada; 3 Department of Pediatrics, Children’s Hospital of Eastern Ontario and Faculty of Medicine, University of Ottawa 6363, Ottawa, Ontario, Canada; 4 CHEO IBD Centre and CHEO Research Institute, Children’s Hospital of Eastern Ontario, Ottawa, Ontario, Canada; Nanchang University, Nanchang, Jiangxi, China

**Keywords:** *Agathobacter*, ulcerative colitis

## Abstract

*Agathobacter rectalis* is a beneficial gut bacterium, known to produce butyrate and thereby enhance gut health. Here, we report the draft genome sequence of *A. rectalis* strain H10.1 isolated from the feces of an individual with ulcerative colitis in clinical remission.

## ANNOUNCEMENT

*Agathobacter (Eubacterium) rectalis* plays a crucial role in colonic resistant starch (RS) fermentation by converting starch breakdown products generated by other bacteria into short-chain fatty acids, especially butyrate (1). *A. rectalis* relative abundance is reduced in patients with inflammatory bowel disease (IBD) as compared to healthy individuals (2). An *A. rectalis* strain was isolated from a fecal sample collected from a participant with the IBD subtype ulcerative colitis in clinical remission. Sample collection was approved by the Research Ethics Board of the Children’s Hospital of Eastern Ontario (REB#20/16E). Following consent, stool was collected at home by the participant and returned to the laboratory in a deoxygenated sterile buffer (1× PBS + 10% wt/vol glycerol, 0.1% wt/vol l-cysteine hydrochloride, pH 7.6). The sample was homogenized to a 20% wt/vol stool slurry, non-bacterial debris was removed by centrifugation, and aliquots were stored at −80°C (3, 4). Bacterial isolation consisted of incubating 300 µL stool slurry anaerobically (5% CO_2_, 5% H_2_, and 90% N_2_) in 1 mL basal medium (5) supplemented with 1.5% ActiStar RT for 4 h at 37°C. After incubation, RS-associated bacteria were isolated as previously described (6). Briefly, the RS-granules underwent multiple cycles of centrifugation at 700 × *g* followed by PBS washes to isolate bacteria specifically attaching to RS-granules. The washed RS-pellet was resuspended in sterile PBS, vortexed, plated on ATCC medium 1703 plates, and incubated anaerobically for 24 h at 37°C. A single colony was picked and purified using three successive isolation streaks on ATCC medium 1703 agar plates under the same conditions and designated as strain H10.1.

The purified H10.1 strain was cultured anaerobically in ATCC medium 1703 broth at 37°C and genomic DNA was extracted using a Quick-DNA HMW kit (ZymoBIOMICS, D6060) according to the manufacturer’s instructions. Unsheared and non-size-selected DNA was used to generate tagmentation libraries in technical triplicate with a Rapid Barcoding Kit (ONT, SQK-RBK114-24) and sequenced on a MinION using a single flow cell (ONT, FLO-MIN114) for 72 h. Subsequent bioinformatic analyses used the default parameters for each program unless otherwise noted. Raw reads were base-called, adapter-trimmed, and demultiplexed using Dorado (v0.9.2, https://github.com/nanoporetech/dorado) with the high accuracy (hac) model to reduce errors. Low-quality reads (*Q*-score < 8) were discarded. The technical replicates were merged, and a total of 370,004 reads (*N*_50_ = 6,800 bp) used for *de-novo* assembly with Flye (v2.9.3-b1797) (7) employing Flye’s built-in error correction module. The draft genome was polished using Medaka (v1.8.1), and the assembly quality was evaluated with QUAST (v5.0.2) (8). Genome completeness was assessed with BUSCO (v5.8.2_cv1) (9).

**Fig 1:**
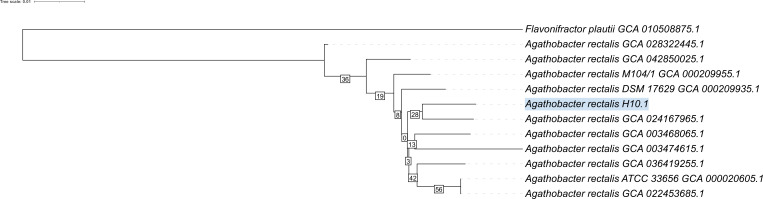
Phylogenetic tree of *Agathobacter rectalis* H10.1 and *Agathobacter rectalis* strains. Tree inferred with FastME 2.1.6.1 (14) from Genome BLAST Distance Phylogeny (GBDP) distances calculated from genome sequences with *Flavonifractor plautii* as an outgroup. The branch lengths are scaled in terms of GBDP distance formula d5. The numbers above branches are GBDP pseudo-bootstrap support values >60% from 100 replications, with an average branch support of 22.8%. The tree was rooted at the midpoint (15).

H10.1’s draft genome consists of four contigs as summarized in Table 1. Genome annotation using NCBI’s prokaryotic genome annotation pipeline (v6.10) (10) revealed 2,966 protein-coding sequences, 78 RNA genes (15 rRNAs, 59 tRNAs, and 4 ncRNAs), and 97 pseudogenes. H10.1 was confirmed as *A. rectalis* with FastANI (v1.34) (11) and shared 96.88% nucleotide identity with the *A. rectalis* ATCC33656 reference genome (NC_012781.1). H10.1’s phylogeny was verified using whole genome taxonomy-based analysis in Type (Strain) Genome Server (12) and visualized using iTOL (v6) (13) (Fig. 1).

**TABLE 1:** Basic genomic features of the *Agathobacter rectalis* H10.1

Parameter	Value
Genome size (bp)	3,233,719
No. of contigs	4
Largest contig (bp)	3,222,615
*N*_50_	3,222,615
GC (%)	41.93
Mean coverage	303
BUSCO completeness (%)	97.1

## Data Availability

The BioProject for *A. rectalis* H10.1 is PRJNA1276980. Raw sequencing reads from the three technical replicates are available in the Sequence Read Archive under accessions SRR34286650, SRR34286649, and SRR34286651. The GenBank accession of the H10.1 draft genome is JBPDJD000000000.
